# Universal design of instruction to enhance learning for university students with visual disabilities

**DOI:** 10.4102/ajod.v12i0.1156

**Published:** 2023-12-20

**Authors:** Jayshree Singh, Sachin Suknunan

**Affiliations:** 1 School of Education, College of Humanities, University of KwaZulu-Natal, Durban, South Africa; 2 Student Services Division, University of KwaZulu-Natal, Durban, South Africa

**Keywords:** disabilities, diversity, higher education, inclusion, students, university, visual disability, universal design of instruction

## Abstract

**Background:**

There has been a steady increase in the number of students with disabilities (SWD) in Higher Education Institutions (HEIs) in South Africa, with a significant number of students having visual disabilities. Equal access remains a key challenge in the classroom setting, thus emphasising a significant gap.

**Objectives:**

This paper capitalised on the significance of this gap and examined the potential of Universal Design of Instruction (UDI) to promote equal access for students with visual disabilities (SWVDs) in the classroom within a university setting in order to maximise learning outcomes.

**Method:**

The study was conducted at the University of KwaZulu-Natal (UKZN), which had approximately 204 SWVDs. This paper draws primarily on the quantitative component of the study. Data collection entailed distributing a questionnaire to all SWVDs. Analysis was conducted using SPSS 21, which produced descriptive and inferential statistics. The theoretical framework of Sen’s Capability Approach in line with the Social Model of Disability was applied.

**Results:**

Findings indicated a lack of UDI in the classroom with very little compliance to all principles, resulting in increased challenges in teaching and learning for SWVDs. The incorporation of UDI in the classroom does have a positive correlation with learning outcomes.

**Conclusion:**

The incorporation of UDI principles can offer a potential design for easier access to teaching and learning to enhance and maximise learning outcomes; alleviate access challenges in the classroom; and address the negative experiences thereof for SWVDs.

**Contribution:**

The study adds value to the scarce body of knowledge on UDI in the classroom for university SWVDs from a learning enhancement perspective.

## Introduction

The numbers of students with disabilities (SWD) admitted to Higher Education Institutions (HEIs) are increasing, thus complementing the Social Model of Disability. However, while efforts are being made to increase accessibility from a physical access perspective, little is known about the potential of the Universal Design of Instruction (UDI) to improve access and achievement in the classroom for SWD. In addition, globally, there is an abundance of research on UDI for students with visual disabilities (SWVDs) in universities in developed countries, yet there remains a paucity of such research and practice within a South African setting. While limited South African research studies do focus on inclusive learning, not many of them focus on UDI in the classroom, which is the essence of the study. Although this is not specifically a study of inclusive education per se, it is an in-depth study to ascertain the applicability of UDI to a Higher Education context. Despite International, National and Higher Education legislation on anti-discriminatory practices, persons with disabilities continue to experience exposure to social and educational exclusion (Subrayen & Suknunan 2019; Dutta 2013 and Rahman 2019). Universal Design of Instruction encourages resourceful and inclusive pedagogy by presenting curriculum design and learning environments which can be fully adapted to accommodate the diversity of SWD at university (Brandt 2011 and Harbour & Maudous 2011). This paper will contribute to the gap in the existing body of knowledge in a South African setting, and the implications of this can inform various policy and practice contributions from any stakeholders in South African HEIs. Africa is a developing continent and it is imperative to identify the need to employ strategies that incorporate UDI in Higher Education, thus placing the country at a competitive advantage in the global sphere. The findings and discussion presented in this article are based on part of the researcher’s PhD study (Singh 2022) that focused primarily on SWVDs in the classroom. The mixed-method study examined the potential of UDI to enhance learning outcomes for SWVDs and counteract the challenges experienced in the classroom that hinder them from academic achievement and the capabilities thereof. Because of the study being large and extensive in nature, this paper draws primarily on results from the quantitative components of the study.

## Literature review

The authors prefer to use the term ‘students with visual disabilities’ as it is more appropriate within the Social Model of disability. Impairment refers to a problem with ‘physical organs’ and thus more aligned to the outdated medical model of disability. The Social Model of Disability positively evolves from the preceding model whereby impairment is seen as a ‘disability’ that can be accommodated through reasonable accommodations that can be implemented for equal access. Visual disability is a generic term used to describe a wide range of visual problems (Rahman 2019). It includes categories such as total blindness, mild and severe cases of visual impairment. The manner in which the learner uses residual vision is the main concern of educators. Based on the educational definition of visual impairment, completely blind refers to severely challenged students who must learn Braille in order to read and write, and low-vision students use their residual vision as a primary sense to deal with visual demands concerning suitable assistive devices (Rahman 2019). Dutta (2013) rationalised that inclusive education should facilitate access to the same information, at the same time and possibly in the same way to promote the involvement of SWVDs in mainstream classroom settings. This paper strongly agrees with Dutta (2013) that SWVDs face barriers to learning and that HEIs should break down these barriers through tactile resources to further promote inclusion.

The Centre for Universal Design (1997) defines the seven principles of UDI as:

- Equitable use: The design must be usable to SWVDs.
- Flexibility in use: The design should accommodate a wide range of individual preferences and abilities.
- Simple and intuitive use: The design must be user-friendly regardless of the user’s experience, knowledge or language skills.
- Perceptible information: The design communicates necessary information effectively to the user, regardless of abilities.
- Tolerance for error: The design caters to and minimises the adverse consequences of accidental or unintended actions.
- Low physical effort: The design can be used efficiently and comfortably and without strain.
- Size and space for approach and use: The design must allow use regardless of the person’s body size, posture, or mobility.

According to Ngubane-Mokiwa (2016:2), the main objective of applying UDI is to ‘promote access, participation and progress’ in the pedagogy for all learners, including SWVDs, by applying its seven principles in the classroom. In a global context, research that entails the possibility of implementation of UDI in the classroom to enhance learning capabilities and outcomes for SWVDs abounds (Haegele & Hodge 2016; Zajadacz 2015 and Burgstahler 2015, 2017, 2018).

A close examination of relevant literature depicted UDI as a relatively new framework in Higher Education. This included studies by Izzo, Murray and Novak (2008); Embry, Parker and Scott (2005) and Rickerson and Deitz (2003). Universal Design of Instruction motivates the development of teaching methods and strategies that are innovative, effective, and efficient (*Higher Education Opportunity Act* [HEOA] 2008). The inclusion of such a definition in the first federal legislation of the United States (HEOA 2008) demonstrated the escalating importance of the inclusion of UDI at HEIs (Izzo 2012). Universal Design of Instruction is a catalyst in bringing about flexibility and creativity to instructional methods, thus allowing SWVDs to acquire knowledge by capitalising on their strengths.

Other studies consulted included those by Singh (2017); Brandt (2011); and Harbour and Maudous (2011), wherein the UDI encouraged an innovative and inclusive pedagogy and valued the diverse range of learners. This was found to be consistent with the Republic of South Africa, Department of Higher Education and Training’s (RSA, DHET 2013) mandate. In addition, the study draws from universal design systems based on the *Higher Education Opportunity Act (2008)* and the United States’ Department of Education’s National Education Technology Plan (2010), where it was emphasised that UDI was a framework that benefited all learners. As such, adherence to the set principles of the universal design approach could propel the university to the next level in the transformation towards greater accessibility for SWVDs.

Organisations such as the Association on Higher Education and Disability (AHEAD) have recognised the importance of UDI (Roberts et al. 2011). The *Higher Education Opportunity Act of 2008* described it as a ‘scientifically valid framework for guiding educational practise’ (Roberts et al. 2011:7). The above legislation consulted supported the study in exploring the potential of the UDI in promoting inclusive learning to enhance learning for SWVDs in the higher education classroom.

Several scientific research outcomes have suggested that progress has been achieved (Bhattacharya 2017; De Montfort University [DMU] 2019; Munene 2017). Inclusive education in high-income countries and many low and middle-income countries have adopted accessibility policies and are reaping the benefits. This study embarked on an extensive global search to improve current perspectives on how to introduce inclusive education practices for SWVDs in the classroom by focusing on the values of diverse societies and their logical co-existence with SWDs. It was observed in studies by DMU (2019), Munene (2017), and Bhattacharya (2017) that the implementation of accessibility standards evolved and they are well-implemented in some countries that appear to be beyond the reach of countries like South Africa because of their limited resources and inadequate enforcement and adherence to policy. To increase the access and independence of SWVDs and all students within the classroom, UDI implementation is appropriate in creating instructional goals, methods, materials, and assessments that work for everyone (Black, Weinberg & Brodwin 2014).

Many universities in South Africa, inclusive of University of KwaZulu-Natal (UKZN), still apply outdated teaching and learning methods where the presentation of the lecture is predominately through projector slides and the chalkboard, with the expectation that everybody understands. Hence, universities need to adapt to change because the historic education systems did not meet the requirements for the diverse groups such as the SWVDs they support today. Therefore, this study embarked on the exploration of a highly recognised model that has been implemented in other countries globally to tackle such a problem. The University of Connecticut (Harbour & Maudous 2011) and the University of Washington (Burgstahler 2013) have successfully implemented UDI. However, there is no supporting evidence as to whether or not universities such as UKZN are UDI-compliant in the classroom. The potential of becoming compliant needs to be explored in order to facilitate or maximise learning outcomes for a growing number of SWVDs.

There is an abundance of research on UDI in Higher Education in developed countries, along with other developing countries around the world raising important questions about UDI and its implementation in classrooms and educational systems (Dalton, Mckenzie & Kahonde 2012). Based on such studies, the researcher raises the general question of why there is such a paucity of Universal Design Systems in HEIs within a South African context. Based on various Internet searches via the academic search engine strategies that incorporate UDI in Higher Education in South Africa, these factors have not been adequately researched nor are there similar studies of this nature across the continent of Africa itself. This paper therefore exploited this gap from a quantitative perspective in terms of inclusive learning in the classroom through UDI, with a particular focus on SWVDs.

## Social model of disability

The Social Model takes the view that if a certain disorder cannot be modified, then outside situations need to be adapted or else SWVDs may experience stigmatisation and feel of less value to society if seen only from the perspective of their dysfunction (Zajadacz 2015). The Social Model of Disability could provide possibilities to create an all-inclusive learning environment that promotes equivalent opportunities for all (Shava 2008). Accordingly, this study drew on the fundamental elements that the Social Model presents on the removal of barriers and its role in increasing the quality of life for SWVDs. The Capability Approach aligns with the Social Model in that the lack of resources can in itself be the catalyst of impairment and/or disability (Mitra 2006). The lack of resources is what causes the disability, which draws similarities with the Social Model that sees disability as a social construct. Disability is not centred on the individual but on the social environment that imposes disability by the way SWVDs are unnecessarily isolated and excluded from full participation in society (Mitra 2006).

## Sen’s Capability Approach

Sen’s Capability Approach was found to be a highly applicable model for the implementation of UDI to maximise learning outcomes in the classroom as it focuses on promoting equal opportunities and equal participation in the classroom, increasing educational development and transformation, increasing autonomy and providing opportunities to achieve. According to Sen’s Capability Approach, capability is understood as a practical opportunity and functioning is the actual achievement of the individual (Mitra 2006). Functionings are states of ‘being and doing’ such as being well-nourished, having shelter and education. In using the capability of education, SWVDs can achieve the function of being valued and contributing members of their society. As a result, experiencing educational equity and being granted the opportunity to participate equally in society lead to an improved quality of life, which is central to the Capability Approach (Schiemer 2017). Broderick (2018) explained that capabilities represent the innate potential of each individual to achieve various outcomes. If SWVDs are provided with the necessary resources, they will be able to function efficiently and produce the desired outcome or achievements such as reading, writing, or communication. This study proposes to enhance capabilities by introducing UDI and embracing the view that human development was a ‘participatory and dynamic process’ that was not primarily concerned with basic need satisfaction (Alkire 2010:5).

Respect for human diversity and equal opportunities are other central tenets of the Capability Approach as they focus on achieving justice by expanding an individual’s capabilities (Broderick 2018). Broderick (2018) and Mitra (2006) share the view that deprivation of opportunities because of a failure to provide reasonable accommodation results in disability and constitutes a form of discrimination. Furthermore, Article 24 of the United Nations Convention on the Rights of Persons with Disabilities (United Nations Convention on the Rights of Persons with Disabilities [UNCRPD] 2006) focused on system changes which involved reasonable accommodations and effective student-centred support for SWVDs (Broderick 2018). Therefore, this study finds the Capability Approach appropriate as it identifies that inclusive education and educational equity are leading approaches to empower SWVDs to live a life they value (Schiemer 2017).

## Research methods and design

The UKZN became the chosen location for the study. The institution has the highest enrolled SWD in the country and hence provided an ideal environment for a study of this nature to be conducted. At the time of the study, the institution had a total of 204 SWVDs (Disability Services Unit 2019). The study adopted a mixed method approach, including both qualitative interviews and quantitative surveys. The quantitative approach involved all SWVDs across the university. The data-collection process involved the administration of questionnaires to these students in appropriate formats for increased accessibility and understandability. As such, questionnaires were made available to all SWVDs via an online tool known as Google Forms^®^. The response rate for the quantitative component was 21 respondents, which contributed to almost 10% of the total population of SWVDs at the institution.

### Data-collection methods and instruments

The research instruments were pilot tested by the Disability Information Access Officer, who is also blind. This added value to the process as he validated the research instruments and tested screen-reading capability. Furthermore, it ensured that the questionnaire was accessible and readable via screen-reading software to SWVDs. The first page of the questionnaire provided SWVDs with an overview of UDI which outlined the seven principles thereof. Upon understanding the objectives of the study, SWVDs were encouraged to respond to the survey questionnaire. The same students also participated in the qualitative component of the study and were already familiarised with UDI in conversations with the researcher/writer.

### Data analysis

The study instrument was developed around the research questions and theoretical frameworks. A questionnaire built on Likert scales was used to extract data through closed-ended questions (Zohrabi 2013). The data were analysed and interpreted using applicable quantitative techniques via SPSS version 21, such as reliability testing, descriptive statistics, and correlation analysis.

### Ethical considerations

All procedures performed in studies involving human participants are in accordance with the ethical standards of the institution. The research was conducted in line with UKZN’s ethical standards for research that involved obtaining ethical clearance from the Humanities and Social Sciences Research Ethics Committee. Ethics consent was received on 03 April 2020. The ethics approval number is [HSSREC/00000872/2019]. The gatekeeper’s letter was obtained, granting permission to conduct research at the UKZN. In addition, informed consent forms ensured voluntary participation. Anonymity and confidentiality were strictly maintained during the data-collection process and the researcher ensured no physical, emotional, psychological, or reputational damage to the participants.

## Findings and discussions

Reliability and validity were discussed as aspects of precision where Cronbach Alpha scores indicated a high degree of reliability. Collectively, the overall Cronbach Alpha score was above 0.7, indicating a high degree of reliability (Lakshmi & Mohideen 2013).

### Biographical attributes

The biographical attributes of the respondents indicated 38% males and 62% females. This indicates a high number of female SWVDs embarking on their studies at the university, which complies with the Women Empowerment and Gender Equality Bill (2013) (section 9 of the Constitution of the Republic of South Africa 1996).

### The nature of disability

**FIGURE 1:**
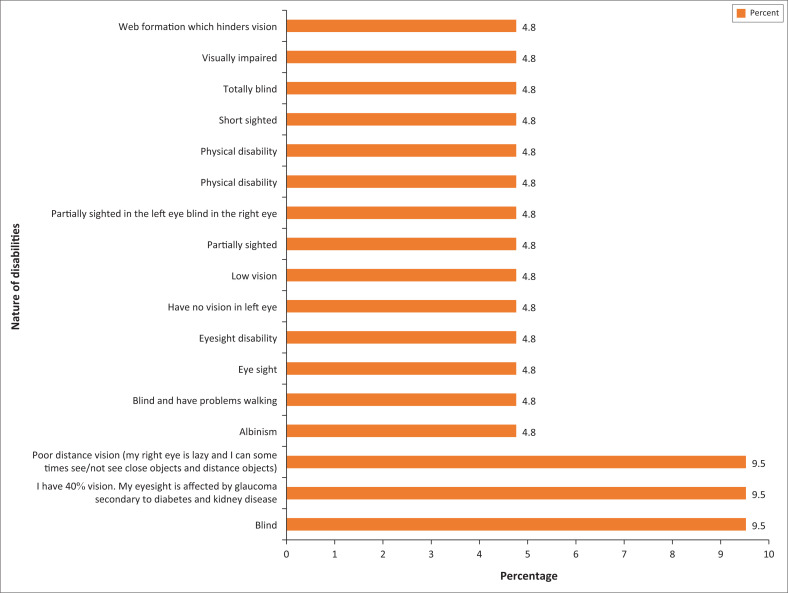
Graphical presentation of the nature of disabilities at University of KwaZulu-Natal.

There was an almost equal distribution of various disabilities related to visual impairment reported by all 21 students (Figure 1). This added value thereby indicating the variety of visual disabilities experienced by students. Hence these students would be able to provide a first-hand account of their experiences.

### Campus and college

There was good distribution across all campuses. However, the majority of participants were from the Howard College and Edgewood, which is in accordance with statistics from the UKZN Disability Support Unit (2021). The majority of the students were from the College of Humanities. This is a logical finding as according to the UKZN Disability Support Unit (2021), the majority of SWVDs are from the Humanities College.

### Year of study

There was a fair distribution in terms of year of study, thereby providing a multi-pronged perspective from respondents in various levels of study.

### The rate of University of KwaZulu-Natal’s compliance to the principles of Universal Design of Instruction in their classroom environment

On a scale of 1–10, where 1 is very poor/non-compliance and 10 is extremely compliant, respondents were asked to rate UKZN’s compliance with the principles of UDI in their classroom environment. Statistics indicate that 13 of 21 respondents gave a ranking of 5 and below for UDI compliance in the classroom, thus indicating a lack of UDI in the classroom.

### Current learning experiences in the classroom

**FIGURE 2:**
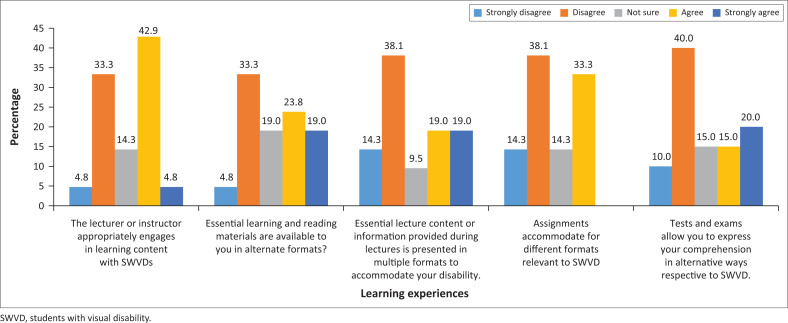
Current learning experiences in the classroom.

Figure 2 indicated that the current learning experiences of SWVDs were fraught with difficulties.

Over 60% of SWVDs indicated a lack of UDI compliance. Baheta and Rabenstein (2018) explained that UDI principles provided multiple modes of learning to support students with and without disabilities in their learning experiences. The lack of UDI in the classroom has resulted in various challenges and this contributes to the overall negative experiences of SWVDs, as reflected by an almost equal agreement and disagreement that the lecturer/instructor appropriately engages in learning content with SWVDs (Figure 2).

Respondents primarily reflected uncertainty and disagreement with essential learning and reading materials being available to them in alternate formats or essential lecture content or information during lectures presented in multiple formats to accommodate their disability. Assignments also did not make accommodations for different formats relevant to the needs of SWVDs, and neither were tests and exams conducted in a way that allowed them to express their comprehension in alternative ways. As UDI implementation involved an adjustment to formats that can improve constraints and access to course content, embracing a new educational perspective aligned with the seven principles of UDI will in turn promote an all-inclusive classroom environment that will increase the satisfaction of the needs of SWVDs and enhance academic achievements (Dutta 2013; Munene 2017). In addition to descriptive statistics, some inferential statistics showed positive relationships affiliated with UDI and enhanced classroom learning for SWVDs. There was a positive correlation between UKZN’s compliance to the principles of UDI and current learning experiences in the classroom (0.447*, *p* < 0.05). This implies that the more UKZN complies with the principles of UDI, the better the learning experiences of SWVDs can be.

The positive correlation revealed that UDI compliance will allow for flexibility in instruction, thereby overcoming barriers and improving learning experiences in the classroom for SWVDs (Heylighen 2014 and Dutta 2013).

### Current challenges in learning for students with visual disabilities in the classroom

Relating to the negative experiences mentioned above, this section described the current challenges experienced by SWVDs in the classroom.

The majority of SWVDs were experiencing difficulty engaging with the lecturers, who do not understand, and have problems adapting to the course content and current support provided.

**FIGURE 3:**
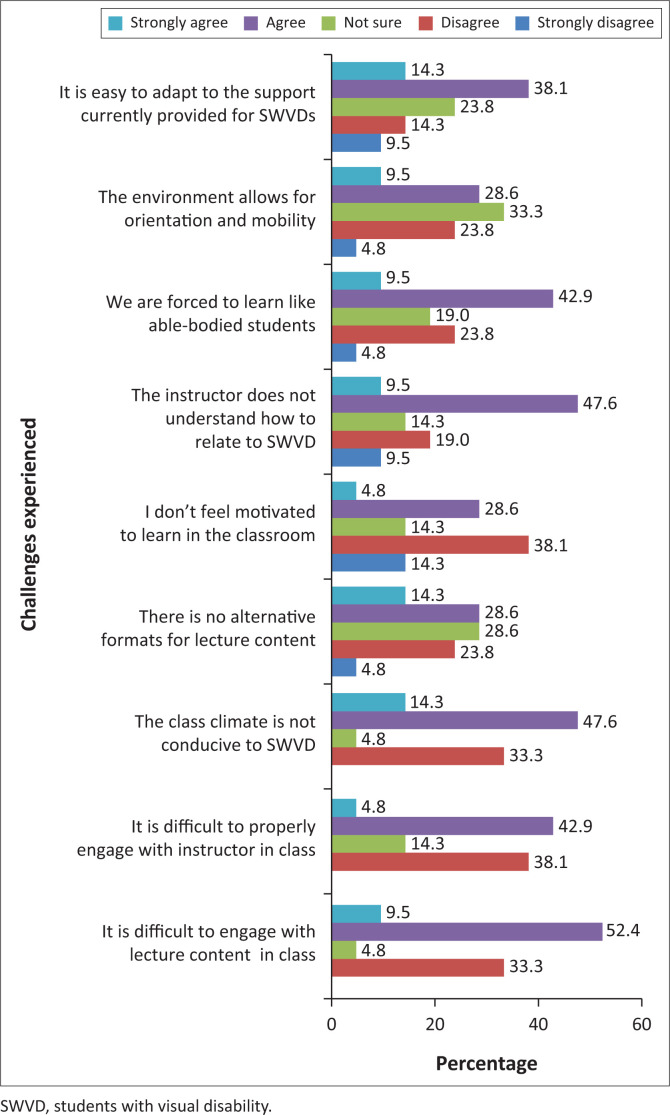
Current challenges experienced by students with visual disabilities in the classroom.

Furthermore, they were forced to learn like able-bodied students, lacked motivation, and found it difficult to engage with the lecture content because of a classroom climate that is not conducive to SWVDs (Figure 3). Black et al. (2014) agreed with Heylighen (2014) that in order to increase access and the independence of SWVDs at university, the process was heavily reliant on accessible systems such as UDI. This was because of the fact that UDI provides a variety of instructional methods and learning techniques, as well as knowledge of how to properly include SWVDs through an understanding of how to provide appropriate accommodations, curriculum, and choices in instruction, in addition to traditional lectures, to create a more inclusive environment.

The inferential findings revealed an inverse relationship between compliance with UDI and current challenges (–0.435*, *p* < 0.05). This implied that an increase in UDI compliance will decrease challenges in the teaching and learning of SWVDs in the classroom. At present, Figure 3 indicated a lack of UDI compliance. Hence the seven principles of UDI are not being used to enhance classroom activities for SWVDs, nor is it used to make education more accessible. If UDI is not effectively applied within the classroom, there can be no positive correlation or relationship and this will adversely impact on challenges that SWVDs experience in the current classroom environment.

### The rate at which the seven principles of Universal Design of Instruction are being met in the classroom

Because of the current challenges and negative experiences listed earlier, it was clear that the seven principles of UDI were not being met in the classroom at the university. This was further confirmed by students.

**FIGURE 4:**
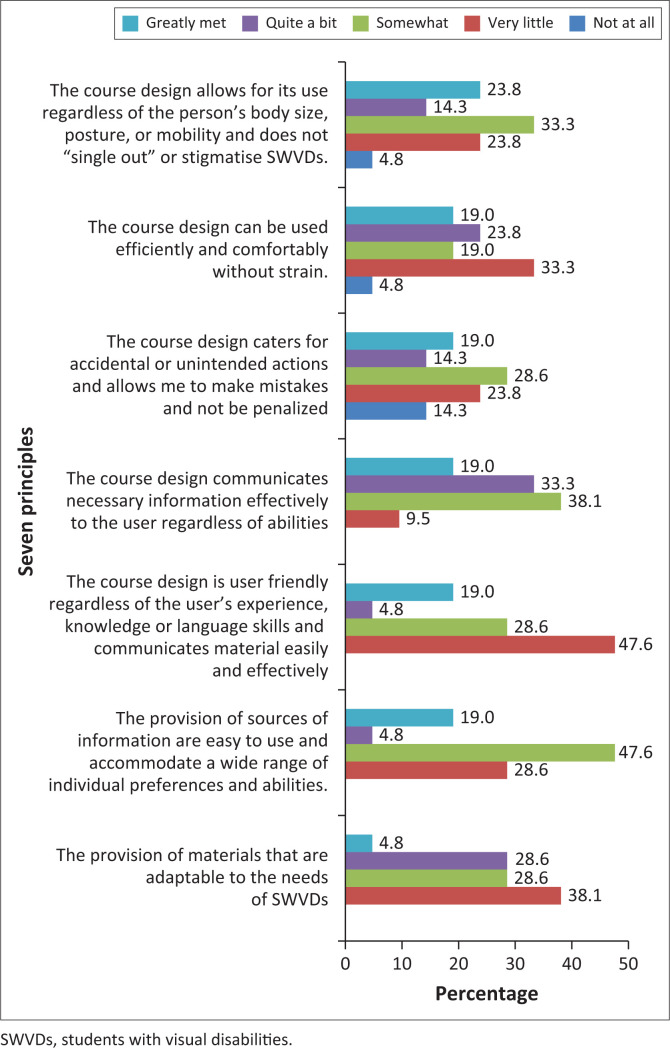
The rate at which the seven principles of Universal Design of Instruction are being met in the classroom.

The majority respondents indicated ‘somewhat to very little’ compliance for all principles (Figure 4). Hence, one can deduce that there was a lack of adaptable study materials, and the course design did not accommodate for the variety of needs of SWVDs and could not be used efficiently and effectively. This collectively indicates poor applicability of UDI principles in the classroom for SWVDs, thereby creating a challenging learning environment. Rahman (2019) and Dutta (2013) concurred that there is no direct correlation between intelligence and visual impairment, and that SWVDs have the same range of intelligence and abilities as sighted students, except that they face additional barriers that may affect their learning. Rahman (2019) argued that problems emerge because of limited opportunities and an education system that is not adapted to their needs. Universal Design of Instruction strategies address these inequities by enhancing the quality of higher education by prioritising equity and the equitable participation of SWVDs in the classroom, as well as by exploring a new balance in teaching and curriculum development (Majoko 2018; Tomozii & Topală 2014).

There was a positive correlation between UKZN’s compliance with the principles of UDI and current learning experiences in the classroom (0.447*, *p* < 0.05). This implied that the more UKZN complies with the principles of UDI, the better the learning experiences of SWVDs can be. Black et al. (2014) claimed that non-compliance with the UDI principles was attributed to a lack of knowledge of how to properly include SWVDs through an understanding of how to provide appropriate accommodations, curriculum, class materials, and choices in instruction that create barriers to their education. Compliance will allow for flexibility in instruction, overcoming barriers and improving learning experiences in the classroom that will aid in improving SWVDs’ learning experience and assist them to adapt to the different situations they may face at university.

### Implementation of Universal Design of Instruction to facilitate or maximise learning outcomes for students with visual disabilities in the classroom

This section examined whether the implementation of UDI will facilitate or maximise learning outcomes for SWVDs in the classroom.

**FIGURE 5:**
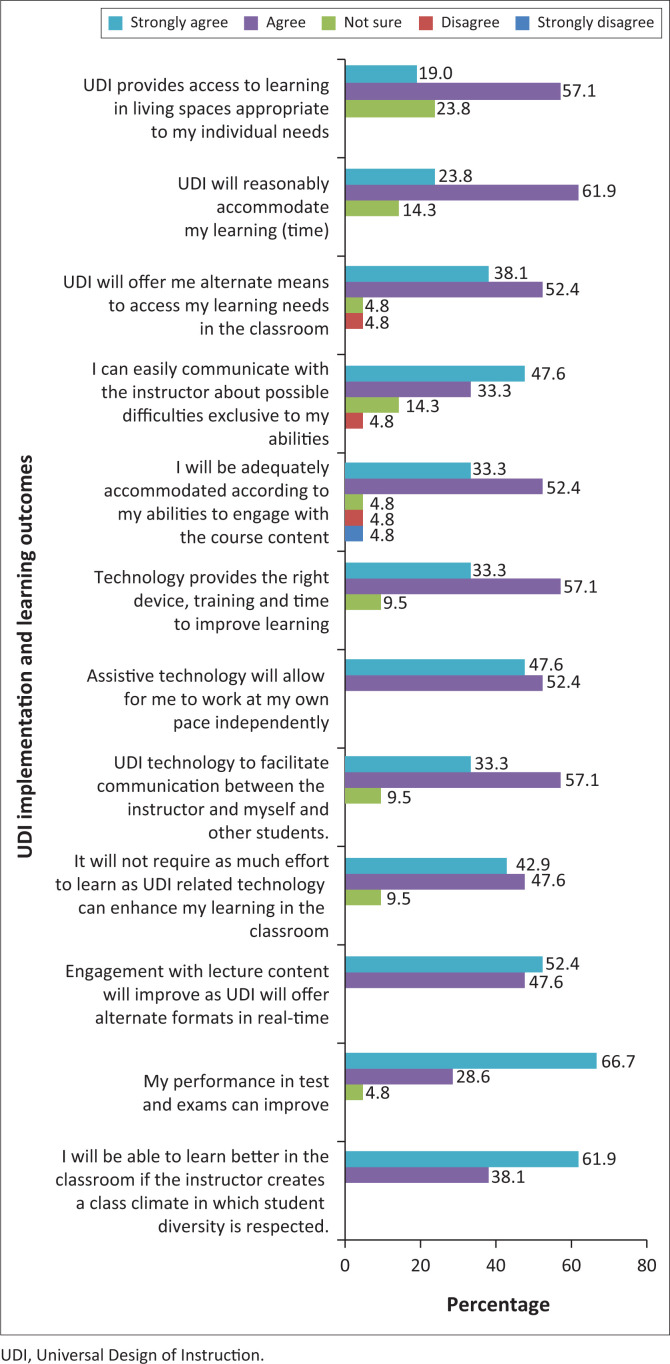
Universal Design of Instruction implementation and learning outcomes.

The majority of the respondents agreed that the implementation of UDI will facilitate or maximise learning outcomes for SWVDs in the classroom (Figure 5). Respondents believed that they will be able to learn better in the classroom if the instructor created a class climate in which student diversity was respected. In addition, it would improve engagement with lecture content as UDI will offer alternate formats in real-time, thus allowing SWVDs to work at their own pace independently. The UDI does not require much effort to learn as UDI-related assistive technology can facilitate communication and enhance learning in the classroom; offers alternate means to access learning needs in the classroom; and provides access to learning in living spaces appropriate to SWVDs’ individual needs as it adequately accommodates them according to their abilities (Schiemer 2017).

There was a significant positive correlation between the implementation of UDI versus facilitates or maximises learning outcomes for SWVDs. This revealed that the implementation of UDI in the classroom can result in maximised learning outcomes for SWVDs (0.537*, *p* < 0.05). Munene (2017) confirmed that the implementation of UDI can be constructively used as a driving mechanism to uphold institutional values and contribute to combating the challenges that SWVDs face, thereby maximising learning outcomes. Hence, it is evident that the implementation of UDI is an appropriate strategy to be applied at the university to ensure that teaching styles, instructional materials, and educational goals are designed and modified to fit the student’s specific learning needs and enhance their visual learning experience (Salleh & Zainal 2010 and Rahman 2019).

### Factors to be considered for the implementation of Universal Design of Instruction to promote inclusive learning for students with visual disabilities in the classroom

**FIGURE 6:**
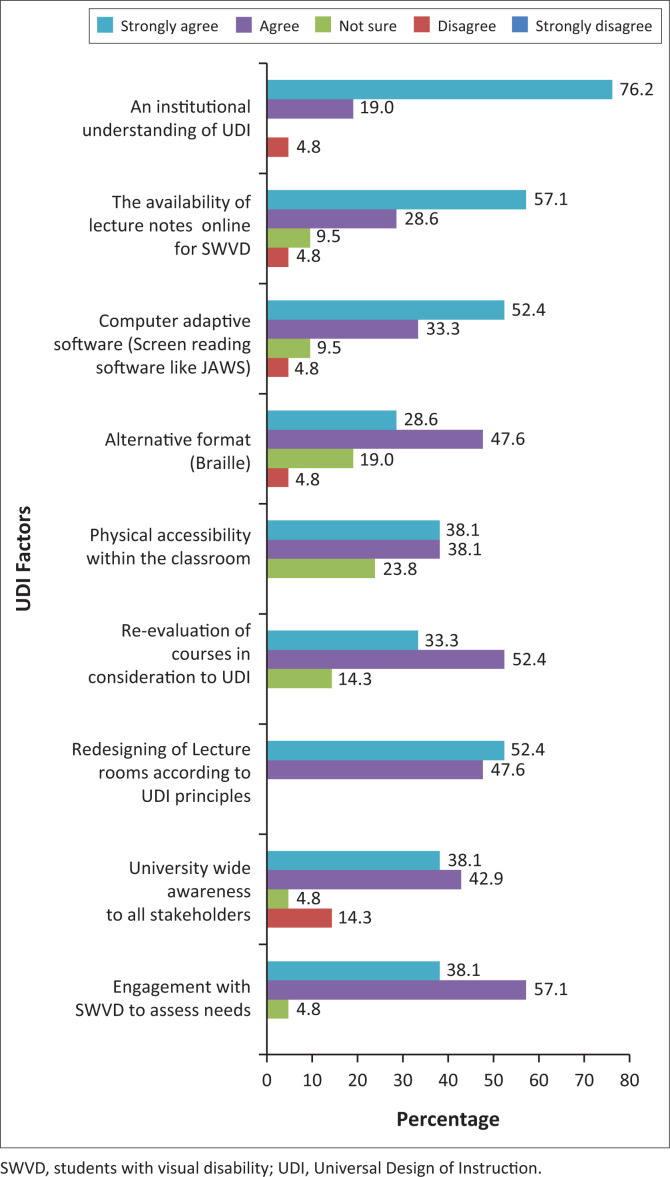
Factors that must be considered for the implementation of Universal Design of Instruction.

For UDI to be implementable in the classroom, it would be dependent on a variety of factors, as supported by respondents (Figure 6).

Several important factors emerged through the study’s findings in conjunction with four highly ranked UDI principles (equitable use, flexibility in use, size and space for approach, and tolerance for error). Figure 6 showed how students rated important factors to be taken into consideration for the implementation of UDI at the university. Firstly, an institutional understanding of UDI was pertinent. Secondly, engagement with SWVDs to assess needs was significant to maximise learning outcomes and create a university-wide awareness that included all stakeholders. The majority were in favour of re-designing lecture rooms and re-evaluating courses in consideration of UDI principles, as the flexibility of a design such as UDI would enable SWVDs to participate in activities promoting interaction among students and instructors. In applying UDI principles, one needs to be cognisant of the diversity of human abilities and conditions and choose the most appropriate design within the context of the classroom (Heylighen 2014). In line with UDI, these factors offer a simplified design for easier access to teaching and learning methods and ensure an adequate and appropriate workspace for students who may require specific arrangements within the classroom. Hewett et al. (2018) expressed that the university needed to transform entrenched attitudes of traditional HE systems and evolve by embracing a new educational perspective such as UDI, which required a period of learning and knowledge acquisition by all stakeholders.

Results further indicated that there is a positive correlation between factors to be considered for the implementation of UDI and implementation of UDI facilitates or maximises learning outcomes (0.475*, *p* < 0.05). It was evident that factors considered for the implementation of UDI to promote inclusive learning will facilitate or maximise learning outcomes for SWVDs in the classroom. Factors may include the need for space to facilitate orientation and mobility, lighting, access to technology, and online access for students who are blind and partially sighted. To ensure that SWVDs will have opportunities to learn, participate and express what they know on an equal level as other students, UDI is an appropriate strategy to be applied at the university (Rahman 2019).

### Sen’s capability approach

**FIGURE 7:**
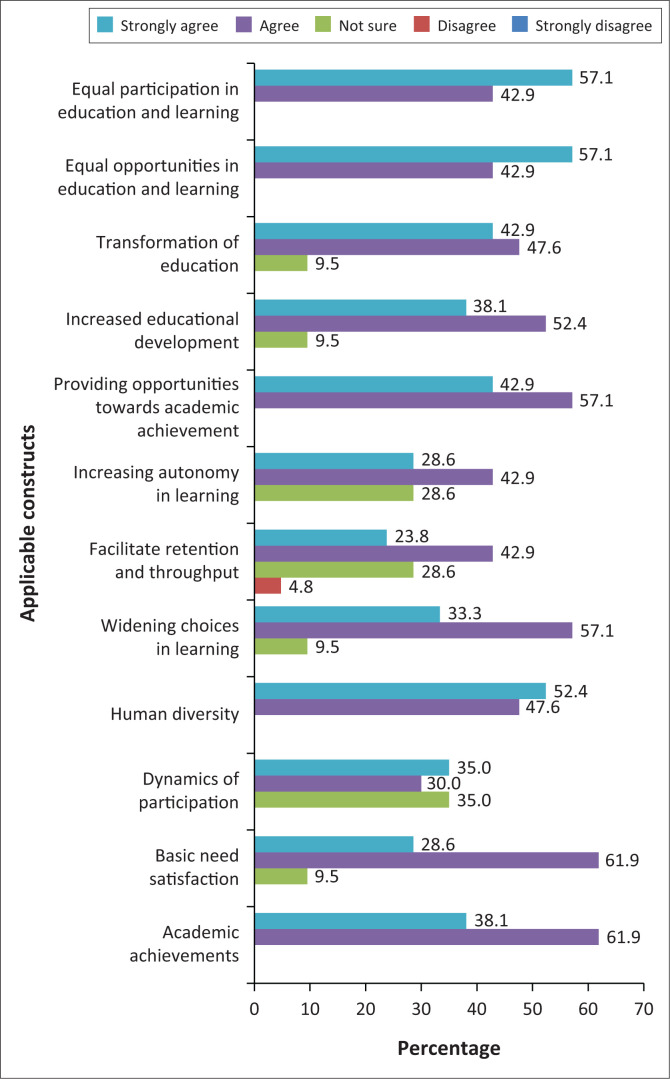
Applicable constructs of Sen’s Capability Approach.

In line with the Social Model of Disability, Sen’s Capability Approach provided favourable grounds for the implementation of UDI, showing significantly higher levels of agreement graphically (Figure 7).

Based on the above statistical outcome, Sen’s Capability Approach is a highly applicable model for the implementation of UDI. The majority of students agreed that the application of UDI in the classroom can maximise learning outcome for SWVDs and enable all stakeholders to work collaboratively in a system within the classroom, promoting shared responsibility through social interaction of SWVDs, lecturers, and other students and participation of SWVDs in all aspects of learning in the classroom. Enhanced capabilities make people autonomous and autonomy leads to possibilities to achieve well-being, quality of life, equity, and equal opportunities for all people (Schiemer 2017). Figure 7 reflected a high level of agreement by SWVDs that enhanced capabilities increased autonomy in learning. The Capability Approach focuses on ensuring equality and developing human potential. This is aligned with the principles of UDI to enhance the capabilities of SWVDs, thereby reducing the consequences of disability and increasing opportunities for SWVDs to satisfy their basic need for quality education (Broderick 2018; Dubois & Trani 2009).

Results revealed a strong positive correlation between applying UDI to enhance academic capabilities and the implementation of UDI in the classroom (0.652**, *p* < 0.05). This indicates that applying UDI in the classroom will enhance the academic capabilities of SWVDs. Students with visual disabilities have existing capabilities that can be enhanced provided that the circumstances or the university environment enables SWVDs to use those capabilities to enhance their functions or actions. The implementation of UDI encourages innovative teaching styles, instructional materials, and educational goals designed and modified to meet the needs of SWVDs. On the contrary, restrictions and limitations of functioning in the classroom that are not compensated for by adaptation of course materials and teaching strategies exacerbate their situation in a mainstream classroom (Dubois & Trani 2009).

The results also indicated that the application of UDI will enhance capabilities which will in turn foster good relationships within the classroom (0.646**, *p* < 0.05). The majority of participants agreed that UDI will enhance capabilities in the classroom through collaborative and reciprocal relationships (give-and-take actions) with the disability coordinator, lecturer, other students, and all relevant stakeholders. Godden and Hsy (2015) agreed that the implementation of UDI is necessary to merge SWVDs into the classroom with able-bodied students, encouraging mutually beneficial relationships. This can further enhance learning capabilities of SWVDs.

## Conclusion

This paper aimed to examine the potential of the UDI in the classroom within a higher education setting to promote equal access for SWVDs in the classroom and facilitate or maximise learning outcomes. In relation to these findings, the study conveyed the potential for inclusive educational practices through the UDI, which can alleviate access challenges in the classroom and address the negative experiences thereof for SWVDs. Both descriptive and inferential findings support the need for the implementation of the UDI in the classroom. Correlations further imply that the increase in the use of the UDI can have a directly proportional relationship towards enhancing and maximising learning outcomes for SWVDs. Therefore, re-conceptualising current teaching and learning practices in line with UDI principles is highly recommended. Furthermore, a move towards UDI will support Sen’s Capability model, which is in line with the Social Model of Disability.
